# Transition-Metal- and Nitrogen-Doped Carbide-Derived Carbon/Carbon Nanotube Composites as Cathode Catalysts for Anion-Exchange Membrane Fuel Cells

**DOI:** 10.1021/acscatal.0c03511

**Published:** 2021-01-28

**Authors:** Jaana Lilloja, Elo Kibena-Põldsepp, Ave Sarapuu, John C. Douglin, Maike Käärik, Jekaterina Kozlova, Päärn Paiste, Arvo Kikas, Jaan Aruväli, Jaan Leis, Väino Sammelselg, Dario R. Dekel, Kaido Tammeveski

**Affiliations:** † Institute of Chemistry, University of Tartu, Ravila 14a, 50411 Tartu, Estonia; ‡ The Wolfson Department of Chemical Engineering, Technion-Israel Institute of Technology, 3200003 Haifa, Israel; § Institute of Physics, University of Tartu, W. Ostwald Str. 1, 50411 Tartu, Estonia; ∥ School of Engineering, Department of Energy Technology, Tallinn University of Technology, Ehitajate tee 5, 19086 Tallinn, Estonia; ⊥ Institute of Ecology and Earth Sciences, University of Tartu, Vanemuise 46, 51014 Tartu, Estonia; # The Nancy & Stephen Grand Technion Energy Program (GTEP), Technion-Israel Institute of Technology, 3200003, Haifa, Israel

**Keywords:** alkaline anion-exchange membrane fuel cell, carbide-derived carbon, carbon nanotubes, electrocatalysis, nitrogen doping, non-precious-metal catalysts, oxygen reduction, stability

## Abstract

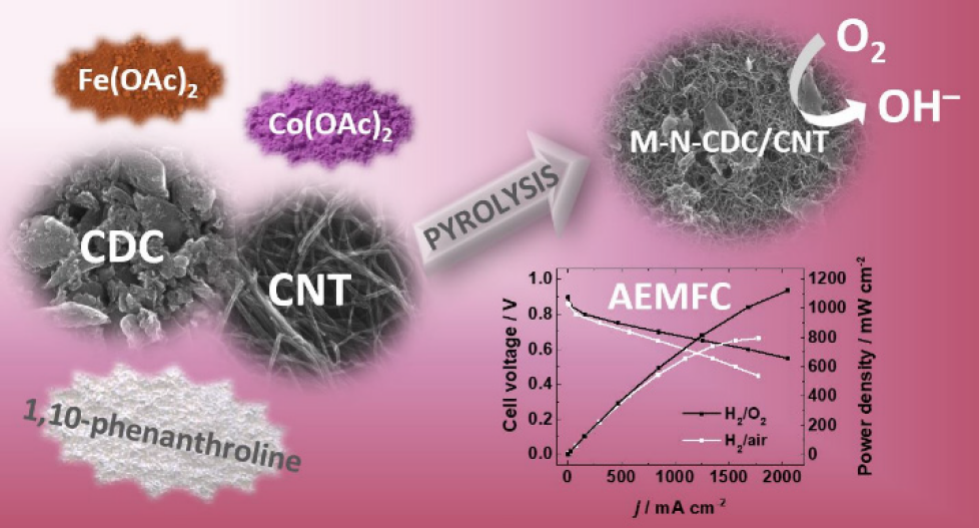

Transition-metal- and nitrogen-codoped carbide-derived carbon/carbon nanotube composites (M-N-CDC/CNT) have been prepared, characterized, and used as cathode catalysts in anion-exchange membrane fuel cells (AEMFCs). As transition metals, cobalt, iron, and a combination of both have been investigated. Metal and nitrogen are doped through a simple high-temperature pyrolysis technique with 1,10-phenanthroline as the N precursor. The physicochemical characterization shows the success of metal and nitrogen doping as well as very similar morphologies and textural properties of all three composite materials. The initial assessment of the oxygen reduction reaction (ORR) activity, employing the rotating ring–disk electrode method, indicates that the M-N-CDC/CNT catalysts exhibit a very good electrocatalytic performance in alkaline media. We find that the formation of HO_2_^–^ species in the ORR catalysts depends on the specific metal composition (Co, Fe, or CoFe). All three materials show excellent stability with a negligible decline in their performance after 10000 consecutive potential cycles. The very good performance of the M-N-CDC/CNT catalyst materials is attributed to the presence of M-N_*x*_ and pyridinic-N moieties as well as both micro- and mesoporous structures. Finally, the catalysts exhibit excellent performance in in situ tests in H_2_/O_2_ AEMFCs, with the CoFe-N-CDC/CNT reaching a current density close to 500 mA cm^–2^ at 0.75 V and a peak power density (*P*_max_) exceeding 1 W cm^–2^. Additional tests show that *P*_max_ reaches 0.8 W cm^–2^ in an H_2_/CO_2_-free air system and that the CoFe-N-CDC/CNT material exhibits good stability under both AEMFC operating conditions.

## 1 Introduction

Energy is an essential part of our lives, which we often take for granted, and with the overall increase in energy consumption, it is causing a significant impact on the environment. The hydrogen economy plays a vital role in reducing this undesired impact, since hydrogen is readily producible, clean, and cost-efficient.^1−4^ A field in which it has gained the most interest is the transportation sector, with intensive research being done for around 20 years, which has led to several automobile makers producing low-temperature polymer electrolyte fuel cell electric vehicles (FCEVs).^5^ Even though the FCEVs have begun to be commercialized, there are still issues related to the use of noble metals, such as platinum, in the fuel cells.^5−7^ In proton-exchange membrane fuel cells (PEMFCs), platinum-based materials are needed on both the anode and cathode electrodes, to withstand the aggressive acidic conditions of the cell. In this regard, anion-exchange membrane fuel cells (AEMFCs) are getting significant attention^8^ as, in principle, their alkaline characteristic offers the opportunity to replace platinum-based catalysts with other, more affordable materials.^9^ For the anodic hydrogen oxidation reaction (HOR), the sluggish kinetics of the HOR in an alkaline medium seems very challenging even for platinum-group-metal (PGM)-based catalysts.^10−17^ For the cathodic oxygen reduction reaction (ORR), while high activity and reasonable durability are presently reached with catalysts based on PGMs,^18−20^ the current focus is to replace them with PGM-free catalysts.

Since the kinetics of the ORR is faster in an alkaline medium than in an acidic medium,^8^ as a replacement of Pt-based ORR materials toward the cathodic ORR, different carbon-based materials have gained attention as well as shown promising performance in AEMFC tests.^21^ However, pure carbonaceous materials lack the necessary electrocatalytic activity^22−25^ and, thus, they need to be modified using non-precious metals^26−32^ or different heteroatoms, such as N,^33−36^ S,^37,38^ P,^39,40^ or their combinations.^41−44^ Among the possible PGM-free electrocatalysts, transition-metal–nitrogen–carbon (M-N-C) type materials are the most promising, since they have shown very good ORR activity as well as superior stability in the half cell in comparison to the platinum-based materials under alkaline conditions.^21,45^

To prepare M-N-C type catalyst materials, the choice of the carbon support is crucial. The selection of carbon materials is wide,^21,46,47^ but among them, the carbide-derived carbons (CDCs) are a great option, as they can reach a very high specific surface area, the reason they are often used in electrochemical capacitors.^48−51^ In addition to this, CDCs may also offer additional advantages; among them, they may impart ultrahigh stability properties to the ORR catalysts. The CDC materials can be produced from numerous carbide sources (e.g. SiC, TiC, ZrC, B_4_C, and Mo_2_C) and by variation of the synthesis conditions (e.g., chlorination temperature), through which the CDC porous structure could be modified and easily reproduced in large quantities.^48,52^ As the CDC materials are mainly microporous, composites of nanocarbons could be used to make materials with both micro- and mesopores. A potential partner for the CDCs would be carbon nanotubes (CNTs) due to their unique tubular structure. In addition to that, the CNTs have excellent resistance to corrosion, a relatively large accessible surface area, and excellent electrical conductivity.^47^

Among the possible transition metals used in M-N-C type ORR catalyst materials, two have been studied the most—cobalt and iron.^21^ In the AEMFC application, cobalt-based materials have shown very good performance,^53−55^ but at the same time, they often have high peroxide production.^55−57^ Iron-based catalysts, on the other hand, are considered to be among the best alternatives to replace platinum for the ORR in the PEMFCs but have often shown moderate activity in the AEMFC,^21,31,58,59^ although there are some exceptions: e.g., work by Santori et al.^60^ In order to reduce the peroxide production on cobalt-based catalysts, an option is to combine two metals into one catalyst material—for example adding iron, since the Fe-N-C materials are considered to bind the ORR intermediates strongly and thus promote a 4e^–^ pathway for the ORR.^56^ The bimetallic catalyst materials have not yet been systematically studied but so far have shown promising performance in half-cell testing, which has been related to the synergetic effects of Fe-N_4_ and Co-N_4_ centers.^61,62^

In this work, three different M-N-C type ORR catalyst materials are synthesized, tested in the half cell using the rotating ring–disk electrode method, and tested in situ in AEMFC devices. As metals, we use Co, Fe, and Co-Fe, to study their independent and combined performance as ORR catalysts. The metal content in the materials is kept low at around 1 wt % to make them more sustainable (especially in terms of cobalt, which is a critical raw material). As a nitrogen source, we use 1,10-phenanthroline, due to its known ability to form complexes with transition-metal salts, leading to the presence of M-N_*x*_ species in the final catalyst material.^63,64^ Metal and nitrogen doping is done via a simple high-temperature pyrolysis method. As the carbon support, we use a composite of CDC and CNTs, to obtain a feasible structure with micro- and mesopores.

## 2 Experimental Section

### 2.1 Catalyst Synthesis

Carbide-derived carbon (CDC), prepared by chlorine treatment of silicon carbide, was acquired from Skeleton Technologies (Estonia). In order to achieve a smaller size of the particles, this CDC material was ball-milled. For the wet ball milling, 200 mg of CDC, 20 mg of polyvinylpyrrolidone (PVP, MW = 40000; Sigma-Aldrich), 3 mL of ethanol, and 20 g zirconium dioxide balls (diameter 0.5 mm) were placed in the grinding bowl. The ball milling was done at 400 rpm for 2 h (4 × 30 min, 5 min cooling breaks). Multiwall carbon nanotubes (CNTs) (NC3150; purity >95%) were purchased from Nanocyl SA (Belgium). As the nitrogen source 1,10-phenanthroline (purity >99%, Acros Organics) was used, and as the metal precursors iron(II) acetate (purity 95%, Sigma-Aldrich) and cobalt(II) acetate (purity >98%, Alfa Aesar) were used.

For the synthesis of the catalyst materials, first metal acetates together with 1,10-phenanthroline (molar ratio 1:6) were dissolved in ethanol. The transition-metal acetates were taken in such an amount that the mass of the metals (Fe or Co) would correspond to 1 wt % of the carbons (CDC and CNT), and in case of dual doping 0.5 wt % of Fe and 0.5 wt % of Co were added. This mixture of metal acetate and 1,10-phenanthroline was treated in an ultrasonic bath (Branson 1510E-MTH, Bransonic) for 30 min. After that, CDC:CNT (1:1 weight ratio) and additional PVP were added to the mixture and sonicated for at least 60 min until a uniform dispersion was achieved. The overall content of PVP corresponded to 1/10 of the weight of the carbons. The prepared liquid dispersion was dried overnight in an oven at 60 °C. The obtained dry blackish powder was then collected and pyrolyzed using a small tube furnace (MTF 12/38/400, Carbolite Ltd.) under an inert atmosphere (N_2_, 99.999%, Linde). The sample was inserted to the heating zone at 800 °C, kept there for 60 min, and then quickly removed, after which the obtained catalyst material was collected. The catalyst materials are designated, according to the metal precursor used for their synthesis (iron(II) acetate, cobalt(II) acetate, or both), as Fe-N-CDC/CNT, Co-N-CDC/CNT, and CoFe-N-CDC/CNT, respectively.

### 2.2 Physicochemical Characterization

For the physicochemical characterization of catalyst materials scanning electron microscopy (SEM) with energy dispersive X-ray analysis (EDX), N_2_ physisorption, X-ray photoelectron spectroscopy (XPS), microwave plasma atomic emission spectroscopy (MP-AES), inductively coupled plasma mass spectrometry (ICP-MS), and X-ray diffraction (XRD) were applied. The detailed experimental descriptions of these techniques can be found in the Supporting Information.

### 2.3 Ex Situ Electrochemical Measurements

The electroreduction of oxygen was studied using the rotating ring–disk electrode (RRDE) method. The electrochemical measurements were done in a three-electrode glass cell containing 0.1 M KOH (purity ≥85%, Sigma-Aldrich) solution at room temperature. Before the experiments, the solutions were saturated with O_2_ (99.999%, Linde) or Ar (99.999%, Linde), and a flow of the respective gas was maintained over the solution during the experiment. As a working electrode, a fixed-disk tip glassy-carbon (GC) disk/Pt ring (Pine Research, USA) electrode was used. The GC with a geometric area of 0.164 cm^2^ was coated by the respective catalysts with a loading of 0.4 mg cm^–2^. The current densities given in this work were normalized to the geometric area of the GC electrode. The preparation of electrodes was done according to a well-established procedure, and the description of it can be found in the Supporting Information. A saturated calomel electrode (SCE) connected through a salt bridge was used as a reference electrode. The potentials presented, however, were converted to the reversible hydrogen electrode (RHE) scale by using the equation *E*_RHE_ = *E*_SCE_ + 0.241 V + 0.059 V × pH. A GC rod (diameter of 3 mm) separated from the working electrode compartment via a glass frit was used as an auxiliary electrode.

Electrochemical experiments were conducted on PGSTAT30 Autolab potentiostat/galvanostat (Eco Chemie BV, The Netherlands) controlled with General Purpose Electrochemical System (GPES) software. An MSRX speed controller and an AFMSRX rotator (Pine Research, USA) were used to conduct the RRDE measurements.

For the detection of a peroxide intermediate (HO_2_^–^) during the RRDE measurements, the Pt ring electrode was kept at a constant potential of 1.55 V. Before the ORR polarization curve was recorded, the electrochemical cleaning of the Pt ring was done by applying at least three potential cycles from 0.05 to 1.65 V at 100 mV s^–1^. The collection efficiency (*N*) of the Pt ring electrode was 0.22, as determined by the hexacyanoferrate(III) reduction reaction. The RRDE data were used to calculate the peroxide yield and the number of electrons transferred. To calculate the percentage yield of HO_2_^–^ formation at the disk electrode, eq 1 was used
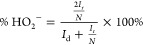
1where *I*_d_ is the disk current, *I*_r_ is the ring current, and *N* is the collection efficiency of the ring electrode. The number of electrons transferred per O_2_ molecule (*n*) was calculated from the RRDE results using eq 2:
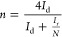
2

For the stability tests, the rotating disk electrode (RDE) method was applied with the working electrode being a GC disk (GC-20SS, Tokai Carbon Ltd., Japan, geometrical area of 0.196 cm^2^) pressed into a Teflon holder and coated with the respective catalyst material. The same catalyst loading was used as in the RRDE measurements. The study of electroreduction of oxygen was done similarly to the RRDE measurements, with the exception of a CTV101 speed controller together with an EDI101 rotator (Radiometer) being used. The stability test was carried out by cycling in the potential range from 1 to 0.6 V at 200 mV s^–1^ for 10000 cycles in O_2_-saturated 0.1 M KOH solution and recording the RDE polarization curves at an electrode rotation rate (ω) of 960 rpm between 1 and −0.2 V at a potential scan rate (ν) of 10 mV s^–1^. In addition, the stability of the bimetallic catalyst (CoFe-N-CDC/CNT) was investigated by applying 30000 potential cycles. After every 10000 cycles, the corresponding data were recorded by the RRDE method.

In addition to the synthesized catalyst materials, an unmodified CDC/CNT sample (ball-milled CDC to CNT ratio of 1:1 by weight) was used as a reference and a commercial 20 wt % Pt catalyst supported on Vulcan carbon XC-72 (E-TEK, Inc.) was employed as a benchmark material for the ORR.

### 2.4 In Situ Anion-Exchange Membrane Fuel Cell Tests

The gas diffusion electrode method was employed to prepare the anode and cathode electrodes for AEMFC testing, following the general procedures previously reported elsewhere.^53,65^ For the anode ink, PtRu/C catalyst (Alfa Aesar, 40% Pt and 20% Ru on carbon black, HiSPEC 10000) was combined with an anion-exchange ionomer consisting of cross-linked polystyrene functionalized with trimethylamine (Fumatech) and carbon black (Vulcan XC-72) and ground with a mortar and pestle to achieve an ionomer to catalyst ratio of 20:80. Additional carbon was added to increase the pore volume and avoid flooding. One part of deionized water and nine parts of 2-propanol were added to the mixture and further ground to create a slurry. For the cathode inks, the M-N-CDC/CNT catalysts were prepared similarly to the anode ink, but without the addition of carbon black and with an ionomer to catalyst ratio of 30:70. The inks were sonicated at 100% intensity for 1 h in a Grant XUBA3 ultrasonic bath filled with water and ice to keep the temperature below 10 °C. After sonication, they were sprayed directly onto 5 cm^2^ gas diffusion layers (Toray carbon paper, 060-TGP-H-060 with 5 wt % PTFE wet proofing) with an Iwata HP-TH professional airbrush. The PtRu loading for all anodes was 0.7 ± 0.05 mg_PtRu_ cm^–2^, while the optimized metal loading was <0.01 mg cm^–2^ for all of the M-N-CDC/CNT cathodes. The cathode loading was kept very low, so to make them more sustainable (especially in terms of cobalt).

The electrodes, along with a 12.25 cm^2^ piece of the radiation-grafted poly(ethylene-cotetrafluoroethylene)-based AEM (Prof. John Varcoe, Surrey, UK),^66,67^ (ETFE) film containing covalently bonded benzyltrimethylammonium (BTMA) head groups (ion-exchange capacity of 2.11 ± 0.11 mmol g^–1^ and 50 μm hydrated thickness), were immersed in 1 M KOH aqueous solution for 1 h, with solution changes every 20 min, to convert the membrane into its hydroxide form. Three different AEMFCs were assembled in situ between two 5 cm^2^ single-serpentine graphite bipolar flow field plates with Teflon gaskets, giving a gas diffusion layer compression of 24%, and torqued to 4.5 N m. The cells were tested in an 850E Scribner Associates Fuel Cell test station with hydrogen at the anode and oxygen at the cathode. The best-performing cell was then subjected to further analysis by switching the oxidant to CO_2_-free air at the cathode. All of the tests were performed at a cell temperature of 60 °C under a gas flow of 1 LPM and 1 barg of back-pressurization on both the anode and the cathode.

## 3 Results and Discussion

### 3.1 Physicochemical Characterization of M-N-CDC/CNT Catalysts

**Figure 1:**
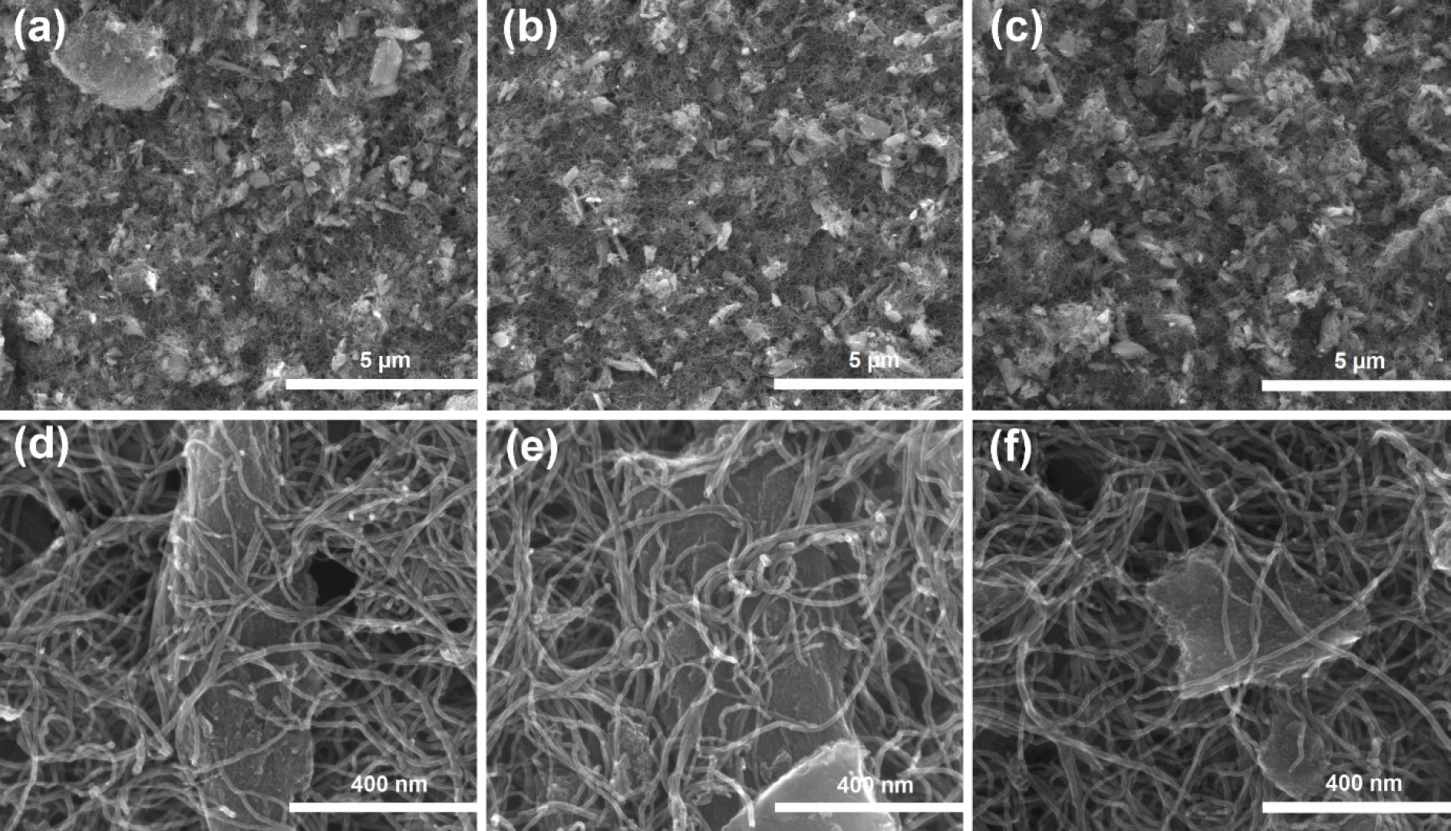
SEM micrographs with lower (a–c) and higher magnification (d–f) for Fe-N-CDC/CNT (a, d), Co-N-CDC/CNT (b, e) and CoFe-N-CDC/CNT (c, f).

The SEM technique was used to study the morphology of the prepared materials. SEM micrographs are shown in Figure 1. Large-scale micrographs (Figure 1a–c) show the relatively homogeneous distribution of CNTs and CDC grains, which vary in size. The CNTs have formed a network between the microporous CDC grains and thus give the material both meso- and macropores. This type of network could be useful in terms of mass transport in the catalyst layer of an AEMFC, mainly at high current densities. Additionally, the CNTs are covering the CDC particles and are themselves aligned in different directions, with some of them being bundled or curled up (Figure 1d–f). As expected, no noticeable differences in the morphology of the materials prepared using different transition metals can be seen.

**Figure 2:**
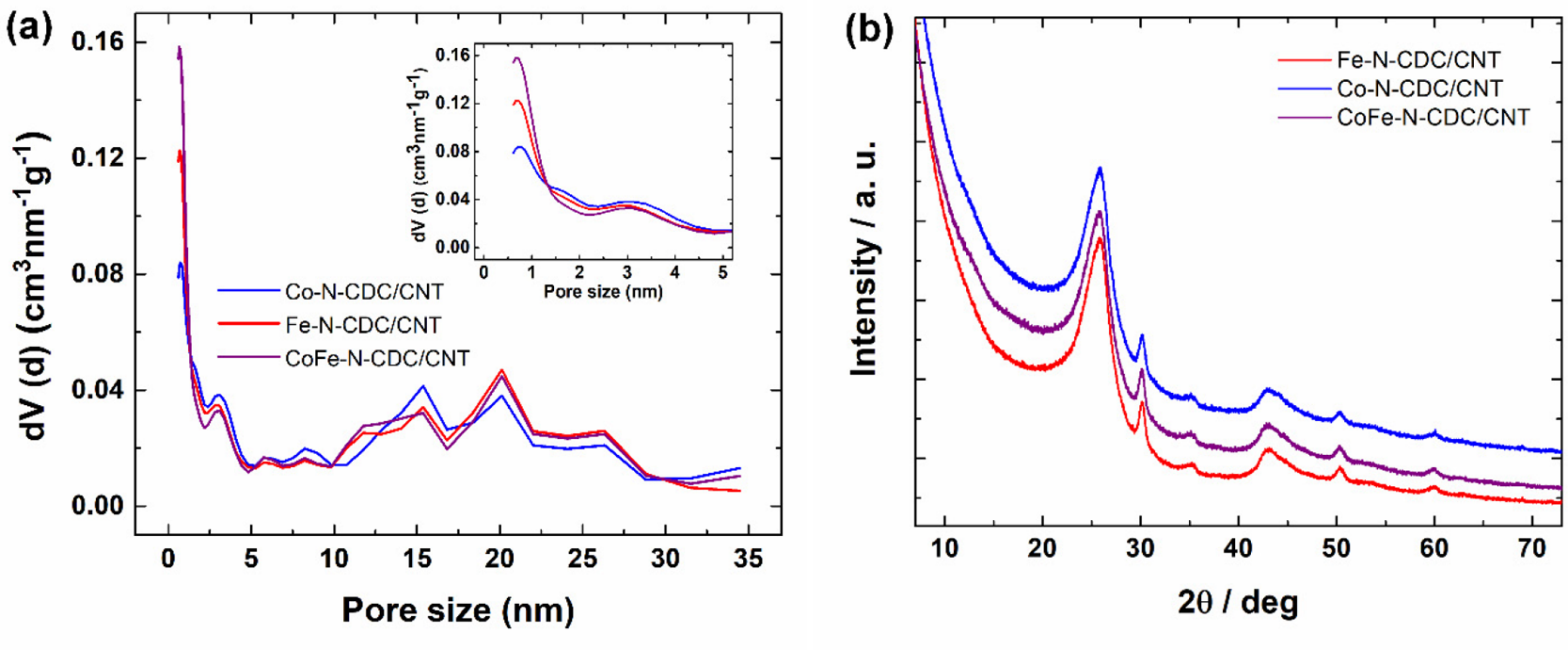
(a) Pore size distributions and (b) XRD patterns for the M-N-CDC/CNT materials.

N_2_ physisorption studies were conducted to analyze the textural properties of prepared M-N-CDC/CNT materials. The results are summarized in Table 1, Figure 2a, and Figure S1. The shape of the isotherms (Figure S1) corresponds to a combination of types I and II with H3 hysteresis according to IUPAC,^68^ which indicates micromesoporous materials with a relatively small proportion of micropores. The specific surface area (SSA) of all catalyst materials was around 400 m^2^ g^–1^. As CDC and CNT were used in equal amounts in weight, then it should be noted that the initial CDC (prior to the ball milling) and the commercial CNTs had SSAs of 1363 and 416 m^2^ g^–1^,^69^ respectively. All three M-N-CDC/CNT catalyst materials exhibit similar porous structures with the total pore volume (*V*_tot_) being 0.5–0.6 cm^3^ g^–1^ and the micropore volume (*V*_micro_) being around 0.1 cm^3^ g^–1^, which means that different metal dopants have similar effects on the textural properties of the materials. The N_2_ physisorption results indicate that the M-N-CDC/CNT materials have both micro- and mesopores present, which could be beneficial in the AEMFC application.^70^

**Table 1:** Specific Surface Area (SSA), Total Pore Volume (*V*_tot_), and Micropore Volume (*V*_micro_) for M-N-CDC/CNT Samples

catalyst material	SSA (m^2^ g^–1^)	*V*_tot_ (cm^3^ g^–1^)	*V*_micro_ (cm^3^ g^–1^)
Fe-N-CDC/CNT	404	0.58	0.11
Co-N-CDC/CNT	393	0.53	0.09
CoFe-N-CDC/CNT	399	0.58	0.12

The XRD patterns for M-N-CDC/CNT materials are shown in Figure 2b. As can be seen in Figure 2b, all three materials exhibit characteristic graphitic carbon peaks at 2θ ≈ 26.2–26.6° and at 2θ ≈ 41–46°. The carbon part of the materials is composed of both 2H and 3R graphite, which characterize the different stacking sequences of graphene layers. The first carbon peak (∼26.5°) corresponds to the reflections of (002) 2H and (111) 3R graphite, while the second (∼46°) corresponds to (100) and (101) 2H as well as (010) and (110) 3R.^71^ The wide first peak with a visible shoulder at lower angles indicates that the materials have a rather heterogenic and disordered structure together with the presence of unlinked graphitic layers. The somewhat disordered structure, as well as the relatively high 3R graphite content, indicates that the M-N-CDC/CNT materials contain a considerable amount of defects.^72^ With regard to the composition of the materials, the XRD analysis showed a contamination with ZrO_2_ (diffraction peaks at ca. 30, 50, and 60°) from the ball milling and the SiC (small peak at ca. 35°) from the starting carbide for the CDC, both in small quantities (<1 wt %), as also shown by the SEM-EDX technique (Table S1). It is not possible to confirm the existence of crystalline Fe or Co species in the materials due to their low metal content and overlapping of possible peaks with the large peak of graphite at 2θ ≈ 46°.

The bulk concentration of iron and cobalt was determined using the MP-AES method, and the results are shown in Table 2. The overall metal content (Fe or Co) was in the range of 1.0–1.2 wt %, which is close to the nominal value calculated from the amount of metal acetates added in the synthesis of the catalysts. It should be noted that the catalyst materials also had some residual metals (not from doping), which originate mainly from the CNTs’ growing substrates (Table S2) and are located mainly inside the carbon nanotubes. It is expected that these particles hidden inside the materials do not affect the electrocatalytic activity, since the CNTs alone have shown relatively poor performance toward the ORR.^73,74^ To evaluate the distribution of metals on the carbon support, the SEM-EDX mapping was applied and the results (Figure S2) indicate that the metals are distributed more or less homogeneously in the CoFe-N-CDC/CNT material with no large agglomerates being visible.

**Table 2:** Surface Elemental Composition of M-N-CDC/CNT Materials as Determined by XPS Analysis and Bulk Metal Composition as Determined by MP-AES

	surface elemental composition (atom %)						bulk metal composition (wt %)	
catalyst	C	O	N	Zr	Fe	Co	Fe	Co
Fe-N-CDC/CNT	96.1	2.7	1.1	0.1	0.1		0.981 ± 0.008	0.049 ± 0.001
Co-N-CDC/CNT	95.9	2.4	1.4	0.1		0.2	0.146 ± 0.004	1.084 ± 0.012
CoFe-N-CDC/CNT	95.5	2.6	1.4	0.1	0.1	0.1	0.620 ± 0.017	0.608 ± 0.008

The XPS results (Table 2 and Figure S3) indicate that in the case of all three samples C, N, O, and Zr, as well as the added respective metals (Fe, Co, or both), are present on the surface. The most prominent elements are carbon, oxygen, and nitrogen, as expected. The nitrogen content was 1.1 atom % for Fe-N-CDC/CNT and 1.4 atom % for Co-N-CDC/CNT and CoFe-N-CDC/CNT, indicating that the doping with nitrogen has been successful for all of the prepared catalyst materials.

**Figure 3:**
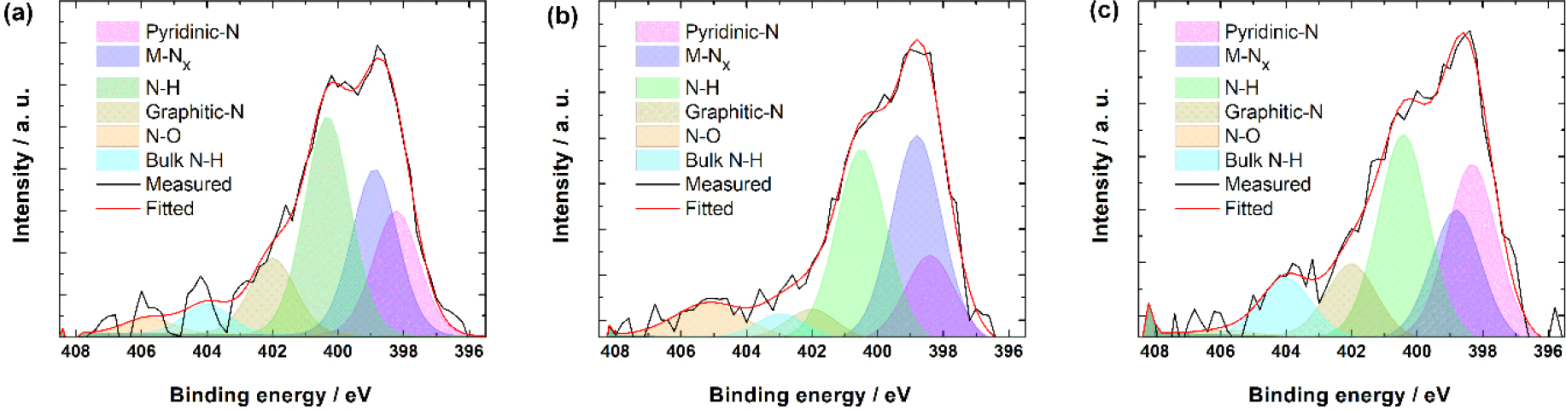
N 1s high-resolution XPS patterns for (a) Fe-N-CDC/CNT, (b) Co-N-CDC/CNT, and (c) CoFe-N-CDC/CNT samples.

As nitrogen is the dopant used, then it is important to determine the type of N species present in the prepared catalysts’ surface. Detailed XPS spectra in the N 1s region were deconvoluted into six peaks corresponding to various N species (Figure 3). The distribution of these species in relative concentrations is shown in Table 3. In all three samples, hydrogenated-N (N–H; includes pyrrolic-N and hydrogenated pyridine), pyridinic-N, and metal-coordinated N (M-N_*x*_) were the most prominent species. In alkaline media, pyridinic-N and M-N_*x*_ are often deemed as the most active sites toward the ORR,^75−79^ and together they make up approximately half of the nitrogen moieties. As N-H moieties are very prominent in the M-N-CDC/CNT materials, there are studies available suggesting that pyrrolic-N is the most active site instead.^80,81^ Also, an alternative option is that the coexistence of different active nitrogen species could be the key to high electrocatalytic activity. For example, Kabir et al. have claimed that the first step of the ORR is HO_2_^–^ formation on graphitic-N, which is then followed by the second step, where pyridinic-N is responsible for the OH^–^ production and the N-H probably catalyzes the 2 × 2e^–^ oxygen electroreduction.^82^

**Table 3:** Relative Concentrations (%) of N Species on M-N-CDC/CNT Materials by XPS Analysis

N species	Fe-N-CDC/CNT	Co-N-CDC/CNT	CoFe-N-CDC/CNT
pyridinic-N	20.0	14.6	27.1
M-N_*x*_	25.7	35.0	20.0
N-H	34.3	32.8	31.4
graphitic-N	12.4	5.1	11.4
N-O	2.9	8.0	0.7
bulk N-H	4.8	4.4	9.3

### 3.2 Oxygen Reduction Reaction (ORR) on M-N-CDC/CNT Catalysts

**Figure 4:**
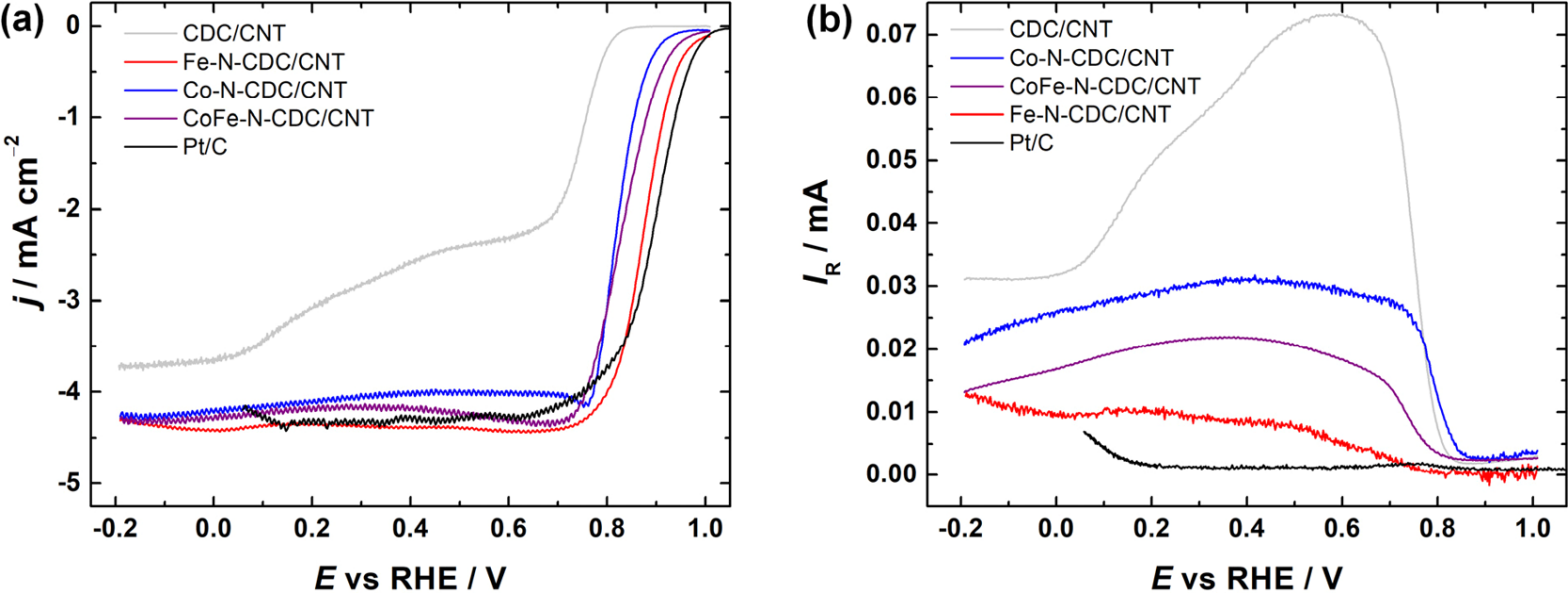
RRDE results for the ORR in O_2_-saturated 0.1 M KOH solution on CDC/CNT, M-N-CDC/CNT, and Pt/C catalysts. (a) disk current densities and (b) ring currents. ω = 960 rpm, ν = 10 mV s^–1^.

In order to give an initial assessment of the prepared catalyst materials’ electrocatalytic activity toward the ORR, the RRDE method was employed. The half-cell tests were conducted in O_2_-saturated 0.1 M KOH aqueous solution at 960 rpm. The corresponding disk current densities and ring currents can be seen in Figure 4. For all three M-N-CDC/CNT catalyst materials, the shapes of the polarization curves were similar: a single oxygen reduction wave along with a clearly defined diffusion-limited current plateau (Figure 4a). The onset potentials of O_2_ reduction (*E*_onset_, the potential at which the ORR current density reaches −0.1 mA cm^–2^) were 0.99, 0.93, and 0.96 V vs. RHE for Fe-N-CDC/CNT, Co-N-CDC/CNT, and CoFe-N-CDC/CNT, respectively. The half-wave potentials (*E*_1/2_) were in the same order: 0.86, 0.82, and 0.83 V. As references, both undoped CDC/CNT materials and commercial Pt/C (20 wt %) were also tested. It is clear that doping with nitrogen and transition metals is useful, since the CDC/CNT composite exhibits two O_2_ reduction waves and a lower *E*_onset_ of 0.80 V. The commercial Pt/C catalyst (loading of 0.08 mg_Pt_ cm^–2^), showed slightly better ORR performance, with the *E*_onset_ and *E*_1/2_ values being 1.00 and 0.89 V, respectively.

These results show that the catalyst with low cobalt content, Co-N-CDC/CNT, has the lowest electrocatalytic activity and the addition of iron (CoFe-N-CDC/CNT) improves the onset potential. The Fe-N-CDC/CNT material itself showed the best performance in terms of both *E*_onset_ and *E*_1/2_.

**Figure 5:**
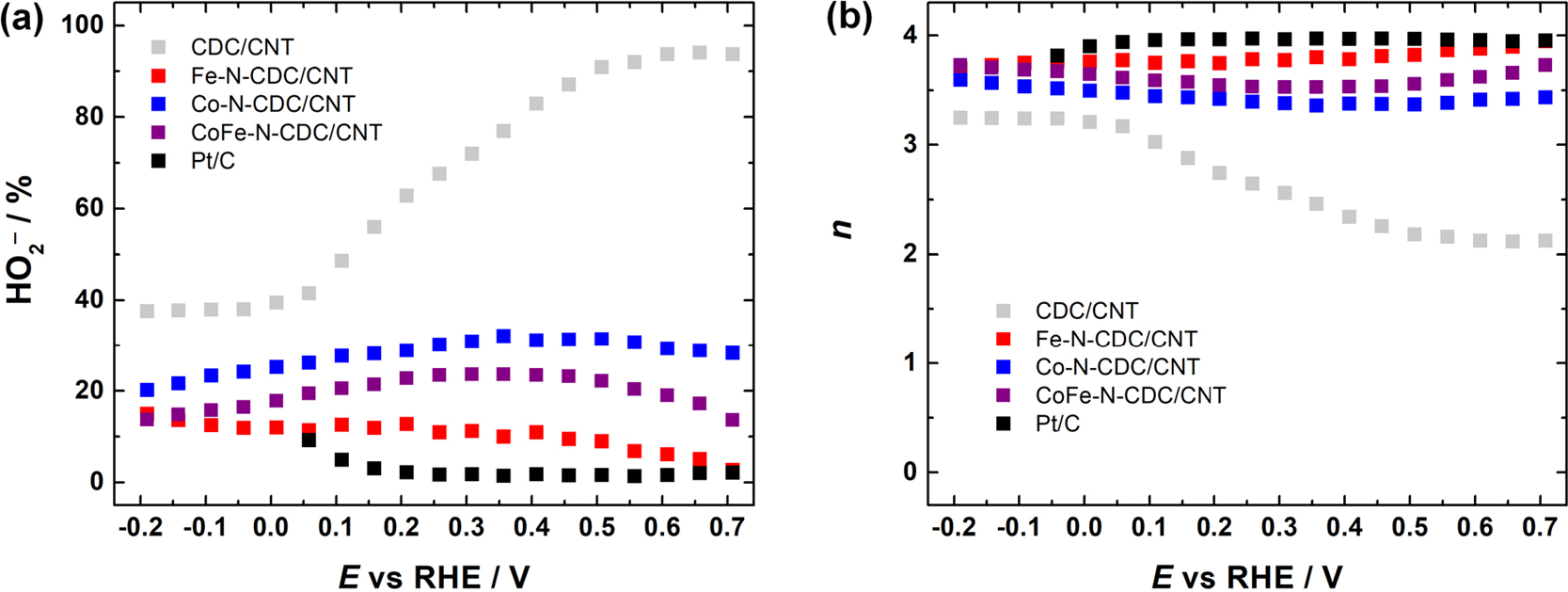
(a) Yield of HO_2_^–^ formation and (b) value of *n* as a function of potential for oxygen reduction on CDC/CNT, M-N-CDC/CNT, and Pt/C catalysts in O_2_-saturated 0.1 M KOH solution. Data are derived from Figure 4.

To study the ORR pathway, the respective ring currents were also collected (Figure 4b). By using the data given in Figure 4 and applying eqs 1 and 2, we calculated the yield of peroxide intermediate and the number of electrons transferred. The yield of HO_2_^–^ formation and *n* as a function of potential are presented in Figure 5. The results show that the undoped CDC/CNT material has the highest peroxide formation, which reaches 90% at higher potentials and decreases to 40% at lower potentials. The commercial Pt/C catalyst has the lowest HO_2_^–^ yield of around 2%, which suggests that a 4e^–^ ORR pathway occurs. In case of M-N-CDC/CNT materials, as expected, the cobalt-based material (Co-N-CDC/CNT) produced the highest amount of HO_2_^–^ (20–30%), which further confirms that cobalt indeed facilitates peroxide production.^56^ The RRDE results also show that the aim of reducing the peroxide production of cobalt-based materials by making bimetallic catalysts has been successful, with the HO_2_^–^ yield being 15–20% in case of CoFe-N-CDC/CNT. The Fe-N-CDC/CNT catalyst showed the least peroxide formation (around 10%). The average *n* values were around 3.5, 3.7, and 3.9 for Co-N-CDC/CNT, CoFe-N-CDC/CNT, and Fe-N-CDC/CNT, respectively. These results indicate that the electroreduction of O_2_ on M-N-CDC/CNT catalyst materials proceeds mostly via a 2 × 2e^–^ pathway, where peroxide forms as an intermediate, which is then further reduced to OH^–^.^83^ These insights are also critical for a further understanding of the degradation mechanisms in AEMFCs, as it has been shown that reactive oxygen species can be generated during AEMFC operation. Indeed, a recent study reported the detection of stable radicals (DMPO-OOH and DMPO-OH) in the cathodes during AEMFC operation.^84^

**Figure 6:**
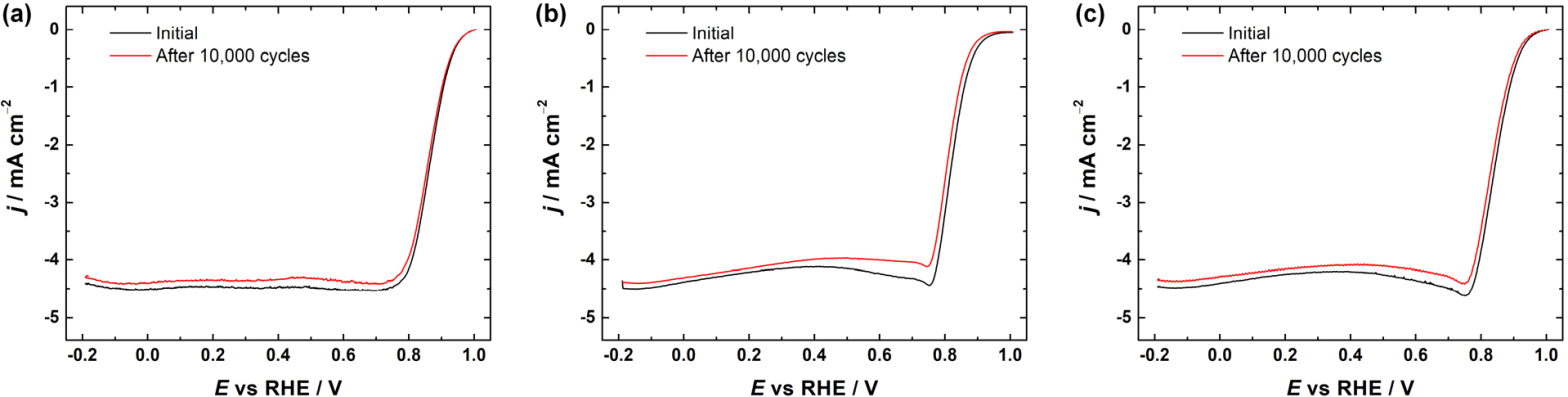
RDE voltammetry curves for ORR on (a) Fe-N-CDC/CNT, (b) Co-N-CDC/CNT, and (c) CoFe-N-CDC/CNT catalyst materials in O_2_-saturated 0.1 M KOH solution before and after 10000 potential cycles (ω = 960 rpm, ν = 10 mV s^–1^).

In addition to high electrocatalytic activity, the catalyst materials must also be durable for long-term applications. To provide some insights into this, stability tests were conducted with all M-N-CDC/CNT catalysts by applying 10000 potential cycles in O_2_-saturated 0.1 M KOH solution. The results are shown in Figure 6. For all three electrocatalysts, the ORR activity keeps the same shape of the polarization curve, suggesting that the catalysts are highly stable in the alkaline medium. Co-N-CDC/CNT had the largest potential shifts, with Δ*E*_onset_ and Δ*E*_1/2_ being 18 and 12 mV, respectively. Fe-N-CDC/CNT and CoFe-N-CDC/CNT, however, showed excellent and very similar stabilities with very small Δ*E*_onset_ (2 and 6 mV) and Δ*E*_1/2_ (6 and 8 mV) values. Additional stability testing was done with the CoFe-N-CDC/CNT catalyst by applying 30000 potential cycles and using the RRDE method. The ORR polarization curves and the yield of HO_2_^–^ generation are shown in Figure S4. These results further indicate that the CoFe-N-CDC/CNT material is very stable with the *E*_1/2_ value changing only by 22 mV after 30000 cycles. The formation of the peroxide remained somewhat the same, although a greater difference, up to 8%, can be seen in the range of 0.7–0.4 V, corresponding well to the changes in the ORR polarization curves.

### 3.3 Anion-Exchange Membrane Fuel Cell Tests

**Figure 7:**
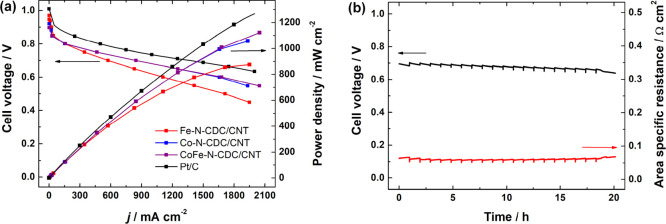
(a) Polarization and power density curves using various cathode catalysts shown in the legend and (b) CoFe-N-CDC/CNT AEMFC in situ stability operation at a constant current density of 0.6 A cm^–2^. All of the tests were performed at a cell temperature of 60 °C under H_2_/O_2_ flows of 1 slpm and 1 barg of back-pressurization on both the anode and cathode.

**Figure 8:**
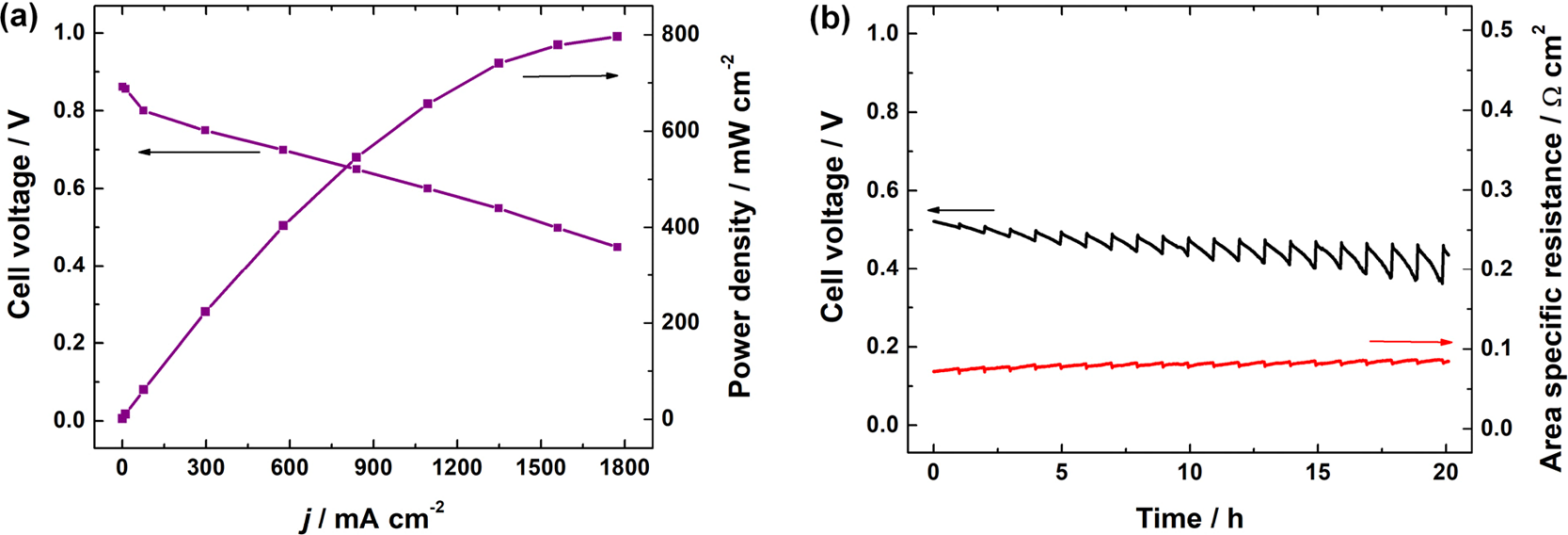
(a) Polarization and power density curves and (b) in situ stability operation at a constant current density load of 0.6 A cm^–2^ using CoFe-N-CDC/CNT as the cathode catalyst. The cathode oxidant was switched to air, and all tests were performed at a cell temperature of 60 °C under H_2_/air flows of 1 slpm and 1 barg of back-pressurization on both anode and cathode.

The H_2_/O_2_ polarization curves of AEMFCs based on the M-N-CDC/CNT ORR catalysts are shown in Figure 7a. It can be clearly seen that the good catalytic activity of CoFe-N-CDC/CNT transferred to very good AEMFC performance. Under the same conditions, at a cell temperature of 60 °C, the CoFe-N-CDC/CNT cathode AEMFC reached a peak power density (*P*_max_) of 1.12 W cm^–2^ and a current density of 0.47 A cm^–2^ at 0.75 V. Despite the RRDE results showing that the Fe-N-CDC/CNT catalyst had the highest ORR activity, it is known in the literature that RRDE results are not always an accurate predictor of fuel cell results, mainly due to the differences in current densities where the RRDE and fuel cells are operated, as well as the mass-transfer-related limitations of fuel cells, which are avoided in the RRDE ideal tests.^85^ To the best of our knowledge, these values for the CoFe-N-CDC/CNT cathode AEMFC are among the highest reported in the literature for precious-metal-free cathode catalysts, as indicated in Table S3. For comparison, a MEA based on a 40 wt % Pt/C cathode catalyst (loading 0.70 mg_Pt_ cm^–2^) tested under similar conditions is included. Each of the precious-metal-free cathode catalysts shows impressive AEMFC performance with the Co-N-CDC/CNT and CoFe-N-CDC/CNT catalysts even approaching that of the MEA using Pt/C (*P*_max_ = 1365 mW cm^–2^). After the polarization curves were acquired, the current density was held constant at 0.6 A cm^–2^ for 20 h to evaluate the voltage stability under the same conditions used to acquire the polarization curve. As evidenced by Table S3, there are not many reported longevity studies for precious-metal-free cathode catalysts operated under H_2_/O_2_. To the best of our knowledge, the longevity data for the CoFe-N-CDC/CNT in Figure 7b exhibits some of the most stable precious-metal-free cathode catalyst data for AEMFCs to date, with a peak to peak loss of 0.03 V after 20 h (1.5 mV h^–1^). When it is borne in mind that the current focus of PGM-free ORR catalysts is to replace PGM catalysts, this stability is encouraging and a step in the direction of ultimately achieving significantly lower voltage degradation rates comparable to those of PGM-catalyzed AEMFCs such as 32 and 15 μV h^–1^, achieved by Peng et al.^86^ and Hassan et al.,^87^ respectively. Given the good performance of CoFe-N-CDC/CNT with H_2_/O_2_, following the 20 h of longevity, the cathode oxidant was switched to CO_2_-free air, and additional polarization curves were captured. As shown in Figure 8a, the CoFe-N-CDC/CNT cell continued to perform well, reaching a *P*_max_ value of 0.80 W cm^–2^ and a current density of 0.30 A cm^–2^ at 0.75 V. Again, these values are among the highest reported in the literature for precious-metal-free cathode catalysts, as summarized in Table S4. A similar current density hold at 0.6 A cm^–2^ for an additional 20 h was performed under H_2_/air for the CoFe-N-CDC/CNT cell, as shown in Figure 8b. The performance after 20 h was still relatively high, with a peak to peak loss of 0.06 V (3 mV h^–1^). The spikes in the cell voltage are most likely due to periodic drying in the cell, as the average relative humidity remained constant at 73% for the duration of the experiment. We postulate that this could have been improved if the dew points were slightly adjusted to ensure that the cell was adequately humidified as explained by Hassan et al.*,*^87^ a thinner membrane was used to increase water back-diffusion from the anode to the cathode creating a more than desirable water gradient inside the fuel cell as supported by Veh et al.*,*^88^ and the cathode catalyst layer (CCL) was coated at a higher loading, making it thicker in order to better attract and retain more back-diffused water and in turn decreasing the overall cell resistance and providing adequate water for ORR and AEM hydration as explained by Gutru et al.*,*^89^ in their recent, comprehensive review on water management strategies in AEMFCs. The H_2_/air longevity data is some of the only data reported in the literature for precious-metal-free cathode catalysts in AEMFCs, as indicated by Table S4.

As mentioned above, the H_2_/O_2_ AEMFC performance of M-N-CDC/CNT catalyst materials is close to those of the best-performing non-precious-metal catalysts reported in the literature: namely, the Co-based material by Peng et al.,^54^ the Fe-based material by Santori et al.,^60^ and Co- and Fe-based materials by Wang et al.^90^ This good performance of M-N-CDC/CNT materials studied herein could be connected to the feasible porous structure with mesopores in addition to the micropores^70^ as well as to the considerable presence of the ORR-active nitrogen moieties: namely, M-N_*x*_ and pyridinic-N.^75,76^ The mesopores are especially needed in the AEMFC operation as the micropores could be blocked by the ionomer ,and thus mesopores are responsible for the mass transport through the catalyst layer.^70^ However, the nitrogen moieties, e.g. pyridinic-N, are needed to improve the chemisorption of oxygen, as nitrogen-induced charge delocalization influences it greatly.^75^ The M-N_*x*_ sites are considered to catalyze the 4e^–^ ORR or the second part of the 2 × 2e^–^ pathway.^78^

The best performance together with excellent H_2_/O_2_ AEMFC stability was obtained with the CoFe-N-CDC/CNT material, which can be related to the synergetic effects of Fe-N_4_ and Co-N_4_ centers, which affect the electronic structure of the carbon network.^61,62^ The same catalyst material also exhibited very good H_2_/air AEMFC performance, being among the best reported (see Table S4). In addition, it should be noted that the CoFe-N-CDC/CNT material contains a very small amount of metals (altogether ca. 1 wt % or <0.01 mg_metal_ cm^–2^ in the AEMFC testing), making it more sustainable. All in all, the CoFe-N-CDC/CNT material is a possible candidate as an ORR electrocatalyst in the AEMFC.

## 4 Conclusion

The preparation of M-N-CDC/CNT catalyst materials using a simple high-temperature pyrolysis method was successful with all three metal compositions, namely with Co, Fe, and Co-Fe. All three catalysts exhibited almost identical morphologies and textural properties, showing both micro- and mesopores. The transition-metal content in the M-N-CDC/CNT catalytic materials was around 1 wt %, according to MP-AES, with the bimetallic CoFe-N-CDC/CNT sample containing ca. 0.5 wt % of both Co and Fe. An XPS analysis proved the success of nitrogen doping, with the respective content being slightly above 1 atom % in all materials, with the M-N_*x*_ and pyridinic-N comprising almost half of the N-moieties present on the surface. The M-N-CDC/CNT catalyst materials exhibit very high electrocatalytic activity for the ORR in alkaline media, with the half-wave potentials being 0.82–0.86 V vs RHE. The electrocatalysts also show excellent stability in the half cell, with a negligible decrease in their performance after 10000 consecutive potential cycles. The HO_2_^–^ formation studies showed that Fe-N-CDC/CNT has the lowest, Co-N-CDC/CNT has the highest, and CoFe-N-CDC/CNT has an intermediate %HO_2_^–^ formation, which indicates that the addition of Fe may improve the performance and stability of Co-based catalyst materials. Finally, very good AEMFC performance was demonstrated for all of the M-N-CDC/CNT catalyst materials, with the CoFe-N-CDC/CNT catalyst performing excellently, reaching peak power densities of 1.12 and 0.80 W cm^–2^ in H_2_/O_2_ and H_2_/air, respectively. In comparison to previous studies using precious-metal-free cathode catalysts, the performance stability of the CoFe-N-CDC/CNT-based AEMFC is among the best. The very good performance of CoFe-N-CDC/CNT materials as ORR electrocatalysts is a result of (i) the presence of ORR-active nitrogen moieties, namely pyridinic-N, (ii) the synergetic effects of Fe-N_4_ and Co-N_4_ centers, and (iii) a feasible porous structure with micro- and mesopores.

## Supporting Information Available

Experimental details of the electrode preparation and physical characterization methods (SEM, SEM-EDX, N_2_ physisorption, XPS, MP-AES, ICP-MS and XRD), results of the catalyst material characterization (N_2_ physisorption, ICP-MS, SEM-EDX, XPS), electrochemical results (RRDE stability test), and a literature comparison of the AEMFC (H_2_/O_2_ and H_2_/CO_2_-free air) testing results (PDF)
