# Supplementary material for: Transition-Metal- and Nitrogen-Doped Carbide-Derived Carbon/Carbon Nanotube Composites as Cathode Catalysts for Anion-Exchange Membrane Fuel Cells
Source: ACS Catal. 2021 Jan 28;11(4):1920–31. doi: 10.1021/acscatal.0c03511 (PMC8744415; doi:10.1021/acscatal.0c03511)
Supplement: Supplementary file 1 — cs0c03511_si_001.pdf [file cs0c03511_si_001.pdf]

Supporting Information

Transition Metal and Nitrogen-Doped Carbide-Derived Carbon/Carbon Nanotube Composites as Cathode Catalysts for Anion-Exchange Membrane Fuel Cells

Jaana Lilloja,[†] Elo Kibena-Põldsepp,[†] Ave Sarapuu,[†] John C. Douglin,[‡] Maike Käärik,[†] Jekaterina Kozlova,[§] Päärn Paiste,[∥,⊥] Arvo Kikas,[§] Jaan Aruväli,[⊥] Jaan Leis,[†] Väino Sammelselg,[†,§] Dario R. Dekel,[*,‡,#] and Kaido Tammeveski[*,†]

[†]Institute of Chemistry, University of Tartu, Ravila 14a, 50411 Tartu, Estonia

[‡]The Wolfson Department of Chemical Engineering, Technion – Israel Institute of Technology, 3200003 Haifa, Israel

[§]Institute of Physics, University of Tartu, W. Ostwald Str. 1, 50411 Tartu, Estonia

[∥]School of Engineering, Department of Energy Technology, Tallinn University of Technology, Ehitajate tee 5, 19086 Tallinn, Estonia

[⊥]Institute of Ecology and Earth Sciences, University of Tartu, Vanemuise 46, 51014 Tartu, Estonia

[#]The Nancy & Stephen Grand Technion Energy Program (GTEP), Technion – Israel Institute of Technology, 3200003, Haifa, Israel

1 EXPERIMENTAL

1.1 Physicochemical Characterization of Catalysts

For scanning electron microscopy (SEM) and X-ray photoelectron spectroscopy (XPS) studies, the catalyst materials were suspended in 2-propanol/Milli-Q water (Millipore, Inc.) (volume ratio 1:1), pipetted onto polished glassy carbon (GC) substrates and dried in an oven at 60 °C. For the SEM experiments, a high-resolution scanning electron microscope (HR-SEM) Helios NanoLab 600 (FEI

[*]Corresponding author. Tel.: +972 77 8871792. Email: dario@technion.ac.il (D. Dekel).
[*]Corresponding author. Tel.: +372 7375168; fax: +372 7375181. Email: kaido.tammeveski@ut.ee (K. Tammeveski).

Company) was employed. Both secondary electron (SE) and back-scattered electron (BSE) modes of SEM were used. SEM images were taken using the Through-the-Lens detector (Fe-containing samples) and Everhart-Thornley detector (Co-containing samples) with an accelerating voltage of 5 or 15 kV. The INCA Energy 350 EDS spectrometer (Oxford Instruments) connected to the microscope was used to assess the elemental composition of the samples and to perform element mapping to observe the distribution of the elements in the sample.

The XPS measurements were conducted using a non-monochromatic twin anode X-ray tube (Thermo XR3E2) with the characteristic energy of 1253.6 eV (Mg K_{α}) and an electron energy analyzer SCIENTA SES 100. All the XPS measurements were performed under ultra-high vacuum. The survey scan was collected using the following parameters: energy range = 1000 to 0 eV, pass energy = 200 eV, step size = 0.5 eV, step duration 0.2 s and number of scans 5. The following parameters for the detailed N 1s spectra were used: energy range = 410–390 eV, pass energy = 200 eV, step size = 0.2 eV, step duration 0.2 s, and number of scans at least 50. The raw data were processed using the Casa XPS software (version 2.3.17). Data processing involved the removal of X-ray satellites, peak fitting (using the Gauss–Lorentz hybrid function (GL 70, Gauss 30 %, Lorentz 70 %) and blend of linear and Shirley-type backgrounds), and calculation of atomic concentrations.

For the estimation of porosity characteristics, the N_2 adsorption/desorption isotherms of the materials were recorded at the boiling temperature of nitrogen using a NovaTouch LX2 analyzer (Quantachrome). Prior to measurements, the samples were degassed in vacuum at 300 °C and backfilled with N_2. The total pore volume (V_{tot}) was measured close to the saturation pressure (P/P_0=0.97). The specific surface area (SSA) and pore size distribution (PSD) were calculated using a quenched solid density functional theory (QSDFT) equilibria model for slit-type pores.

The X-ray diffraction (XRD) analysis was carried out using Bruker D8 Advance diffractometer with Ni filtered Cu Kα radiation and LynxEye line detector. The XRD patterns were collected using a scanning step of 0.0183° 2θ in a range from 5° to 90° and the counting time of 525 s per step. The XRD patterns were analyzed using the full-profile analysis software Topas 6 (Bruker).

The transition metal concentration in the catalyst materials was determined using microwave plasma atomic emission spectroscopy (MP-AES). 10 mg of sample material was dissolved with Anton Paar Multiwave PRO microwave digestion system in NXF100 vessels (PTFE/TFM liner) using an acid mixture of 4 mL of HNO_3 (65%; Carl Roth, ROTIPURAN® Supra) and 2 mL of H_2O_2 (30%; Carl Roth, ROTIPURAN®). Samples were digested one by one at 230 °C and pressures between 45-50 bar. After dissolution, the samples were diluted with 2% HNO_3 solution to obtain metal concentrations of around 5 mg L^{-1} and analyzed using Agilent 4210 MP-AES at analytical wavelengths for Fe 371.993 nm and Co 340.512 nm.

The bulk concentration of metals in starting carbon materials was determined by inductively coupled plasma mass spectrometry (ICP-MS). The samples were dissolved with Anton Par Multiwave PRO microwave digestion system using NXF100 digestion vessels (PTFE-TFM liner) in 8 N rotor, prior to analysis. For the analysis, Agilent 8800 ICP-MS/MS was used.

1.2 Electrode Preparation

For the rotating ring-disk electrode (RRDE) measurements, a fixed-disk tip of GC disk/Pt ring (Pine Research, USA) was used. The geometric area of the GC disk was 0.164 cm^2. The surface of the GC electrode was polished using 1, 0.3, and 0.05 µm alumina powders (Buehler). The surface was cleaned via sonication in Milli-Q water and 2-propanol for 5 min in each solvent.

As an electrode substrate for the rotating disk electrode (RDE) measurements, a GC disk (GC-20SS, Tokai Carbon Ltd., Japan) pressed into Teflon holder was used. The electrode's geometric

area was 0.196 cm^2. The GC disk electrode was polished to a mirror finish using 1 and 0.3 μm alumina slurries (Buehler) and cleaned the same way as for the RRDE experiments.

A catalyst suspension was made using 5 mg of catalyst material dispersed in 490 μL of 2-propanol, 490 μL of Milli-Q water, and 20 μL of Nafion ionomer solution (5 wt%, Sigma-Aldrich). The suspensions were sonicated in an ultrasonic bath for at least 1 h until a uniform dispersion was achieved. A catalyst suspension was pipetted onto the cleaned GC surface, to obtain a catalyst loading of 0.4 mg cm^{-2}, and then allowed to dry in an oven at 60 °C.

2 RESULTS AND DISCUSSION

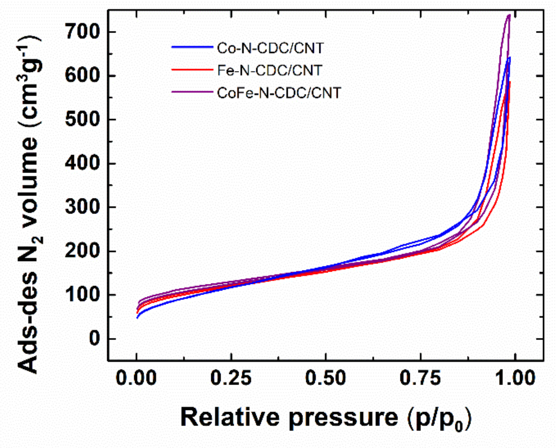

Figure S1. N_2 adsorption-desorption isotherms for M-N-CDC/CNT catalyst materials.

Table S1. Elemental composition (wt%) of the M-N-CDC/CNT materials by SEM-EDX analysis.

Catalyst material	C	N	O	Si	Fe	Co	Zr
Fe-N-CDC/CNT	88.8±0.4	3.9±0.5	4.7±0.3	0.6±0.1	1.0±0.3	0.0±0.0	0.8±0.2
Co-N-CDC/CNT	88.5±0.9	4.4±0.6	4.6±0.7	0.5±0.1	0.1±0.1	1.0±0.2	0.8±0.2
CoFe-N-CDC/CNT	87.0±0.8	4.6±0.5	5.9±0.5	0.6±0.2	0.5±0.2	0.5±0.2	0.7±0.2

Table S2. Metal impurities (wt%) in CNT and CDC materials according to ICP-MS analysis.

	Fe	Co	Al	Ca	Ti	Ni	V
CNT	0.255±0.005	0.106±0.003	0.067±0.001	0.130±0.005	*n.a.*	*n.a.*	*n.a.*
CDC	0.043±0.001	*n.a.*	0.630±0.014	*n.a.*	0.052±0.003	0.032±0.0001	0.100±0.001

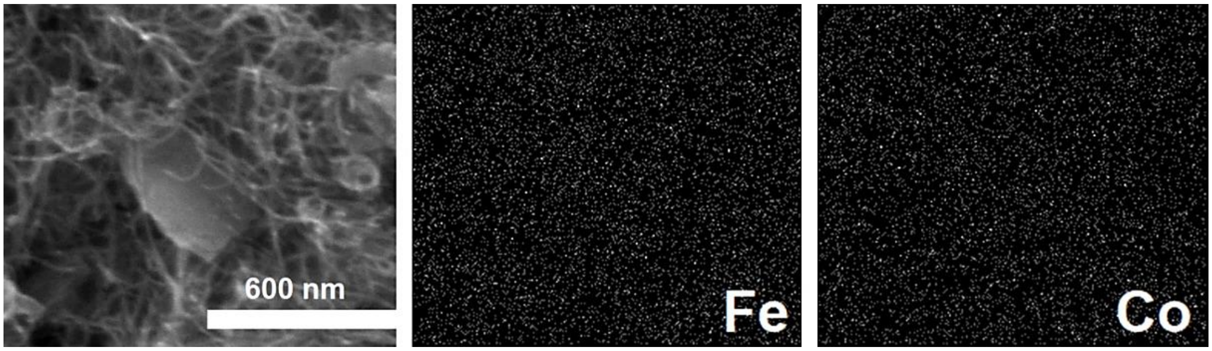

Figure S2. SEM-EDX mapping results for CoFe-N-CDC/CNT sample.

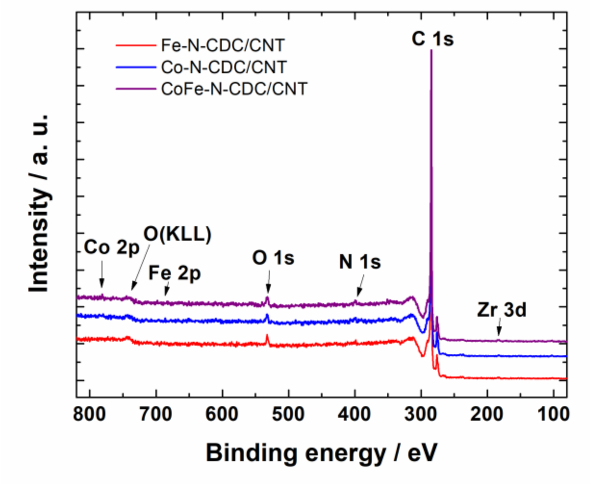

Figure S3. XPS survey spectra for M-N-CDC/CNT samples.

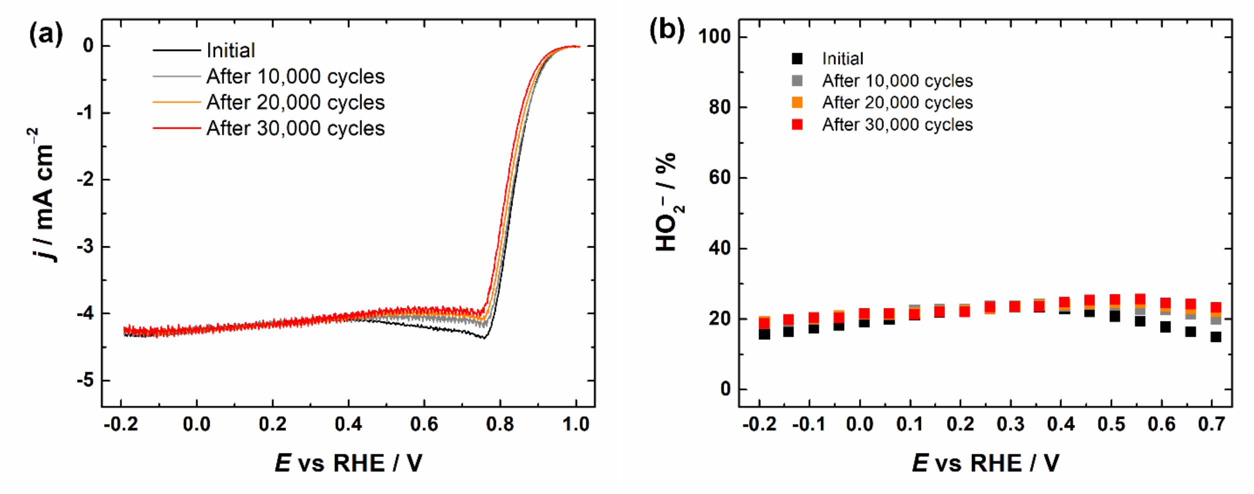

Figure S4. (a) The ORR polarization curves and (b) the yield of HO_2^- formation as a function of potential on CoFe-N-CDC/CNT catalyst materials in O_2-saturated 0.1 M KOH solution before and after 30,000 potential cycles (ω = 960 rpm, v = 10 mV s^{-1}).

Table S3. Comparison of H_2/O_2 AEMFCs using PGM-free cathode electrocatalysts showing performance and longevity results in the literature.

Cathode catalyst	Cathode loading (mg cm^{-2})	Anode loading	Membrane	Cell temp. (ºC)	Anode/Cathode gas flows (LPM)	Anode/Cathode backpressures (barg)	P_{max} (W cm^{-2})	j @ 0.75 V (A cm^{-2})	Decay rate	Ref.
Co-N-CDC/CNT<br>Fe-N-CDC/CNT<br>CoFe-N-CDC/CNT	0.75<br>0.71<br>0.75	0.74 mg_{PtRu} cm^{-2}	ETFE	60	1/1	1/1	1.06<br>0.88<br>1.12	0.47<br>0.35<br>0.47	-<br>-<br>1.5 mV h^{-1}	This work
Ag/C	2.13	0.70 mg_{PtRu} cm^{-2}	HDPE	80	1/1	0/0	1.70	0.25	-	[1]
Ag/C	2.16	0.80 mg_{PtRu} cm^{-2}	LDPE	80	1/1	0/0	1.62	0.29	70 mA h^{-1}	[2]
FeCoPc/C	0.30	0.70 mg_{PtRu} cm^{-2}	LDPE	80	1/1	0/0	1.26	0.20	50 mA h^{-1}	[2]
Fe−N−C	0.90	0.60 mg_{PtRu} cm^{-2}	Unknown	65	1/1	0/0	1.44	0.62	-	[3]
CoFe/VC	2.40	0.70 mg_{PtRu} cm^{-2}	LDPE	70	1/1	0/0	1.35	0.26	-	[4]
Pd-Cu/Vulcan	0.60	0.60 mg_{PtRu} cm^{-2}	ETFE	60	0.2/0.7	0/0	1.1	1	3 mA h^{-1}	[5]
N-C-CoOx	2.40	0.70 mg_{PtRu} cm^{-2}	LDPE	65	Unknown/Unknown	0.8/1.2	1.05	0.49	-	[6]
N-C-CoOx	2.40	0.10 mg_{PtRu} cm^{-2}	LDPE	65	Unknown/Unknown	0.9/1	0.74	0.39	1.29 mV h^{-1}	[6]
FU-N/C	1.00	0.80 mg_{PtRu} cm^{-2}	ETFE	60	1/1	0/0	0.70	0.21	-	[7]
Fe–N_x-CNT/PC	2.00	0.50 mg_{Pt} cm^{-2}	Unknown	80	0.4/1.2	0/0	0.38	0.18	-	[8]
Fe/N/CNT	2.00	0.40 mg_{PtRu} cm^{-2}	*a*QAPS-S_8	60	0.2/0.2	1/1	0.49	0.15	-	[9]
SiCDC/CNT(1:3)/CoPc	0.69	0.73 mg_{PtRu} cm^{-2}	ETFE	60	1/1	0/0	0.47	0.17	-	[10]
NHCS-W	4	0.4 mg_{Pt} cm^{-2}	A201	60	0.2/0/2	1.4/1.4	0.13	0.075	1 mA h^{-1}	[11]
FeCN-S-800	3.0	0.8 mg_{Pt} cm^{-2}	FAA-3	50	Unknown	0/0	0.125	0.01	23.4 mV h^{-1}	[12]
CoNPC-71	4.0	0.5 mg_{Pt} cm^{-2}	A201	60	0.4/0.4	0/0	0.068	0.070	0.2 mA h^{-1}	[13]

Table S4. Comparison of H_2/air AEMFCs using PGM-free cathode electrocatalysts showing performance and longevity results in the literature.

Cathode catalyst	Cathode loading (mg cm^{-2})	Anode loading	Membrane	Cell temp. (ºC)	Anode/Cathode gas flows (LPM)	Anode/Cathode backpressures (barg)	P_{max} (W cm^{-2})	j @ 0.75 V (A cm^{-2})	Decay rate	Ref
CoFe-N-CDC/CNT	0.75	0.74 mg_{PtRu} cm^{-2}	ETFE	60	1/1	1/1	0.80	0.30	3 mV h^{-1}	This work
Ag/C	2.16	0.80 mg_{PtRu} cm^{-2}	LDPE	80	1/1	0/0	0.86	0.10	-	[2]
FeCoPc/C	0.30	0.70 mg_{PtRu} cm^{-2}	LDPE	80	1/1	0/0	0.36	<0.05	-	[2]
CoFe/VC	2.40	0.70 mg_{PtRu} cm^{-2}	LDPE	70	1/1	0.3/1	0.68	0.06	-	[4]
N-C-CoOx	2.40	0.70 mg_{PtRu} cm^{-2}	LDPE	65	Unknown/Unknown	0.8/1.2	1.05	0.49	1.1 mV h^{-1}	[6]
N-C-CoOx	2.40	0.10 mg_{PtRu} cm^{-2}	LDPE	65	Unknown/Unknown	0.9/1	0.74	0.39	-	[6]
Ag/C	1.00	< 150 μg_{Pt} cm^{-2}	PAP-TP-85	95	0.15/0.95	2.5/1.3	0.92	0.5	0.33 mV h^{-1}	[14]

REFERENCES

(1) Wang, L. Q.; Peng, X.; Mustain, W. E.; Varcoe, J. R. Radiation-Grafted Anion-Exchange Membranes: The Switch From Low- to High-Density Polyethylene Leads to Remarkably Enhanced Fuel Cell Performance. *Energy Environ. Sci.* **2019**, *12*, 1575-1579, DOI: 10.1039/c9ee00331b.

(2) Wang, L.; Bellini, M.; Miller, H. A.; Varcoe, J. R. A High Conductivity Ultrathin Anion-Exchange Membrane with 500+ h Alkali Stability for Use in Alkaline Membrane Fuel Cells that Can Achieve 2 W cm^{-2} at 80 C. *J. Mater. Chem. A* **2018**, *6*, 15404-15412, DOI: 10.1039/C8TA04783A.

(3) Firouzjaie, H. A.; Mustain, W. E. Catalytic Advantages, Challenges, and Priorities in Alkaline Membrane Fuel Cells. *ACS Catal.* **2020**, *10*, 225-234, DOI: 10.1021/acscatal.9b03892.

(4) Peng, X.; Kashyap, V.; Ng, B.; Kurungot, S.; Wang, L. Q.; Varcoe, J. R.; Mustain, W. E. High-Performing PGM-Free AEMFC Cathodes from Carbon-Supported Cobalt Ferrite Nanoparticles. *Catalysts* **2019**, *9*, 264, DOI: 10.3390/catal9030264.

(5) Peng, X.; Omasta, T. J.; Roller, J. M.; Mustain, W. E. Highly Active and Durable Pd-Cu Catalysts for Oxygen Reduction in Alkaline Exchange Membrane Fuel Cells. *Front. Energy* **2017**, *11*, 299-309, DOI: 10.1007/s11708-017-0495-1.

(6) Peng, X.; Omasta, T. J.; Magliocca, E.; Wang, L. Q.; Varcoe, J. R.; Mustain, W. E. Nitrogen-Doped Carbon-CoO_x Nanohybrids: A Precious Metal Free Cathode that Exceeds 1.0 W cm^{-2} Peak Power and 100 h Life in Anion-Exchange Membrane Fuel Cells. *Angew. Chem. Int. Ed.* **2019**, *58*, 1046-1051, DOI: 10.1002/anie.201811099.

(7) Lu, Y. X.; Wang, L. Q.; Preuss, K.; Qiao, M.; Titirici, M. M.; Varcoe, J.; Cai, Q. Halloysite-Derived Nitrogen Doped Carbon Electrocatalysts for Anion Exchange Membrane Fuel Cells. *J. Power Sources* **2017**, *372*, 82-90, DOI: 10.1016/j.jpowsour.2017.10.037.

(8) Sa, Y. J.; Seo, D. J.; Woo, J.; Lim, J. T.; Cheon, J. Y.; Yang, S. Y.; Lee, J. M.; Kang, D.; Shin, T. J.; Shin, H. S.; Jeong, H. Y.; Kim, C. S.; Kim, M. G.; Kim, T. Y.; Joo, S. H. A General Approach to Preferential Formation of Active Fe-N-x Sites in Fe-N/C Electrocatalysts for Efficient Oxygen Reduction Reaction. *J. Am. Chem. Soc.* **2016**, *138*, 15046-15056, DOI: 10.1021/jacs.6b09470.

(9) Ren, H.; Wang, Y.; Yang, Y.; Tang, X.; Peng, Y. Q.; Peng, H. Q.; Xiao, L.; Lu, J. T.; Abruna, H. D.; Zhuang, L. Fe/N/C Nanotubes with Atomic Fe Sites: A Highly Active Cathode Catalyst for Alkaline Polymer Electrolyte Fuel Cells. *ACS Catal.* **2017**, *7*, 6485-6492, DOI: 10.1021/acscatal.7b02340.

(10) Praats, R.; Käärik, M.; Kikas, A.; Kisand, V.; Aruväli, J.; Paiste, P.; Merisalu, M.; Sarapuu, A.; Leis, J.; Sammelselg, V.; Douglin, J. C.; Dekel, D. R.; Tammeveski, K. Electroreduction of Oxygen on Cobalt Phthalocyanine-Modified Carbide-Derived Carbon/Carbon Nanotube Composite Catalysts. *J. Solid State Electrochem.* **2021**, *25*, 57-71, DOI: 10.1007/s10008-020-04543-z.

(11) Tong, J.; Ma, W.; Bo, L.; Li, T.; Li, W.; Li, Y.; Zhang, Q. Nitrogen-Doped Hollow Carbon Spheres as Highly Effective Multifunctional Electrocatalysts for Fuel Cells, Zn–Air Batteries, and Water-Splitting Electrolyzers. *J. Power Sources* **2019**, *441*, 227166, DOI: 10.1016/j.jpowsour.2019.227166.

(12) Huang, H. C.; Su, C. Y.; Wang, K. C.; Chen, H. Y.; Chang, Y. C.; Chen, Y. L.; Wu, K. C. W.; Wang, C. H. Nanostructured Cementite/Ferrous Sulfide Encapsulated Carbon with Heteroatoms for Oxygen Reduction in Alkaline Environment. *ACS Sustainable Chem. Eng.* **2019**, *7*, 3185-3194, DOI: 10.1021/acssuschemeng.8b05033.

(13) Sanetuntikul, J.; Hyun, S.; Ganesan, P.; Shanmugam, S. Cobalt and Nitrogen Co-Doped Hierarchically Porous Carbon Nanostructure: A Bifunctional Electrocatalyst for Oxygen Reduction and Evolution Reactions. *J. Mater. Chem. A* **2018**, *6*, 24078-24085, DOI: 10.1039/c8ta08476a.

(14) Wang, J. H.; Zhao, Y.; Setzler, B. P.; Rojas-Carbonell, S.; Ben Yehuda, C.; Amel, A.; Page, M.; Wang, L.; Hu, K.; Shi, L.; Gottesfeld, S.; Xu, B. J.; Yan, Y. S. Poly(Aryl Piperidinium) Membranes and Ionomers for Hydroxide Exchange Membrane Fuel Cells. *Nat. Energy* **2019**, *4*, 392-398, DOI: 10.1038/s41560-019-0372-8.
